# 3D Imaging of Rapidly Spinning Space Targets Based on a Factorization Method

**DOI:** 10.3390/s17020366

**Published:** 2017-02-14

**Authors:** Yanxian Bi, Shaoming Wei, Jun Wang, Shiyi Mao

**Affiliations:** School of Electronic and Information Engineering, Beihang University, Beijing 100191, China; biyanxian@126.com (Y.B.); weishaoming@126.com (S.W.); maoshiyi@buaa.edu.cn (S.M.)

**Keywords:** 3D imaging, spinning target, factorization, scaling

## Abstract

Three-dimensional (3D) imaging of space targets can provide crucial information about the target shape and size, which are significant supports for the application of automatic target classification and recognition. In this paper, a new 3D imaging of space spinning targets via a factorization method is proposed. Firstly, after the translational compensation, the scattering centers two-dimensional (2D) range and range-rate sequence induced by the target spinning is extracted using a high resolution spectral estimation technique. Secondly, measurement data association is implemented to obtain the scattering center trajectory matrix by using a range-Doppler tracker. Then, we use an initial coarse angular velocity to generate the projection matrix, which consists of the scattering centers range and cross-range, and a factorization method is applied iteratively to the projection matrix to estimate the accurate angular velocity. Finally, we use the accurate estimate spinning angular velocity to rescale the projection matrix and the well-scaled target 3D geometry is reconstructed. Compared to the previous literature methods, ambiguity in the spatial axes can be removed by this method. Simulation results have demonstrated the effectiveness and robustness of the proposed method.

## 1. Introduction

Because of its abundant information about the target’s structure and size, three-dimensional (3D) radar imaging of space targets plays a vital role in the space target recognition and classification field. Recently, the research on 3D imaging of space targets, such as space debris, ballistic targets, satellites and so on, has drawn intensive attention [1,2,3,4]. Here by 3D imaging we mean estimating the 3D coordinates of the target scattering centers from the target echoes. Up to now, various techniques have been proposed to generate high resolution 3D images of space targets using wideband inverse synthetic aperture radar (ISAR). 

According to the number of sensors, the 3D imaging technique can be roughly classified into two categories [5]. The first category is interferometric ISAR imaging [2,6,7,8,9]. By using conventional two-dimensional (2D) ISAR imaging algorithms, the target echoes received by the different antennas are processed to form 2D range-Doppler (RD) images. Then the height of the scattering centers is extracted via the phase difference between the 2D images. These conventional 2D imaging algorithms assume the scattering center echo signal energy can be focused in one range and the Doppler resolution cell during the imaging time [8,9]. However, for rapidly spinning targets, there may be migration in the range and Doppler resolution cells, which results in a smeared 2D ISAR image. Therefore, how to achieve well-focused images by the conventional algorithms due to the time-varying range and Doppler information is a challenge [10].

The second one is based on monostatic ISAR, which exploits one-dimensional (1D) range series or 2D range-Doppler image sequences [11,12,13,14,15,16,17] to reconstruct the 3D distribution of the scattering centers. In [1,11,12,13,14,15] the singular value decomposition (SVD) is applied to the 1D range-only matrix to extract the 3D coordinates of scattering centers. This method requires the target to have a 3D rotational motion after the translational motion compensation, which is not applicable to a spinning target. In [16], GRT-CLEAN is proposed to form the 3D image of the spinning target. It is worth mentioning that the 3D images achieved via this algorithm are modified by a scaling factor in the cylindrical coordinate system. Mayhan et al. [17] assumed the target motion parameters are known and proposed the “snapshot” 3D imaging method which employs a sequence of range and range-rate data to extract the scattering centers’ 3D coordinates to obtain unambiguous scattering center coordinates. In a practical scenario, the target motion parameters are usually unknown and need to be estimated before using this method. With the development of the new reconstruction algorithm in the computer vision community [18,19], McFadden [20] and Li et al. [21] applied the factorization method to 2D RD image sequences to obtain the 3D geometry reconstructions based on the 3D-to-2D orthographic projection between the 3D geometry and 2D image sequence. Because of the unknown spinning angular velocity, the reconstructed points also have a certain scale ambiguity in the spatial axes. 

The abovementioned problems in 3D imaging of spinning targets motivate our research. In this paper, a novel 3D imaging algorithm for rapidly spinning space targets based on the factorization method is presented. Compared to currently available methods, it can remove the 3D image scaling ambiguity in the shape matrix. Firstly, after the translational compensation of the echo data, the sequence of the independent 2D high resolution scattering center range and range-rate is extracted using 2D spectral estimation techniques [22]. Compared to the conventional Fourier Transform (FT) method, this technique offers two advantages: (1) it provides enhanced resolution both in range and range-rate; (2) it produces a scattering center trajectory matrix consisting of range and range-rate after implementing the scattering centers correlation developed later in this work. Secondly, the scattering centers association is implemented based on the Kalman filter and minimum Euclidean distance criterion [23]. Thirdly, by using a coarse angular velocity, the scattering centers projection matrix consisting of scattering centers’ range and cross-range is obtained. Then, a more accurate target spinning angular velocity estimate is achieved by applying the factorization method to the projection matrix. After that, the estimate angular velocity is used to rescale the projection matrix and the scaled projection matrix is factorized to obtain the newly accurate angular velocity estimate iteratively. Finally, the accurate angular velocity estimate is used to rescale the projection matrix and then factorize the scaled projection matrix. The advantage of the proposed method is that it achieves a well-scaled 3D image of the target. 

The remainder of this paper is organized as follows: After an introduction, the 3D imaging geometry of the spinning target is introduced in Section 2. Subsequently, Section 3 presents the range and range-rate acquisition process. Then, the scattering center association process is described in Section 4. Section 5 describes the 3D imaging and scaling procedure in detail. The simulation results to verify the effectiveness and robustness of the proposed method are presented in Section 6. Finally, Section 7 draws the conclusion.

## 2. Imaging Geometry

**Figure 1:**
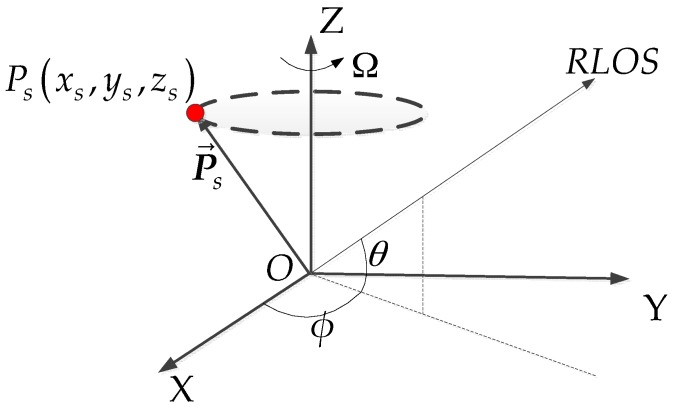
3D imaging geometry of a spinning target.

In this section, the 3D imaging geometry of space spinning targets will be derived and analyzed in detail. For the applications considered here, we assume that the observed space target is spinning around one major axis during the observation interval [16,24]. Figure 1 shows the 3D imaging geometry after the coherent pre-processing and translational compensation [24]. Assume the target local coordinates system is *O−XYZ* and *O* is the origin. The space target spins around the *Z* axis and the angular velocity $\vec{\omega }=\left(0,0,\Omega \right)$. After the translational compensation, the unit vector of the RLOS (radar line of sight) is a constant [4]. Suppose that the azimuth and elevation angle of the RLOS are *φ* and *θ*, respectively. The unit vector of the RLOS can be written as $\hat{n}=\left(\cos\theta \cos\phi ,\cos\phi \sin\phi ,\sin\theta \right)$.

When the radar transmits a high frequency wideband signal, the target can be represented by a reasonable number of fiducial non-coplanar dominant scattering centers. Suppose that the target is rigid and consists of *S* point scattering centers, whose positions are denoted as ${\vec{P}}_{s}={\left(x_{s},y_{s},z_{s}\right)}^{T},\left(s=1,\ldots ,S\right),$ So time instant *t*, after the coherent pre-processing and translational compensation [25], the scattering center instantaneous radial range $r_{s}\left(t\right)$ and range-rate ${\dot{r}}_{s}\left(t\right)$ induced by the spinning motion can be derived as follows:

(1) $$r_{s}\left(t\right)=\hat{n}\cdot {ℜ}_{t}\cdot {\vec{P}}_{s}$$

(2) $${\dot{r}}_{s}\left(t\right)=\hat{n}\cdot \left(\vec{\omega }\times \left({ℜ}_{t}\cdot {\vec{P}}_{s}\right)\right)=\left(\hat{n}\times \vec{\omega }\right)\cdot {ℜ}_{t}\cdot {\vec{P}}_{s}$$

where ${ℜ}_{t}\in {\mathbb{R}}^{3\times 3}$ is called the 3D rotation matrix [26] and here, ${ℜ}_{t}=\left[\begin{array}{ccc} \cos\left(\Omega t\right) & -\sin\left(\Omega t\right) & 0\\ \sin\left(\Omega t\right) & \cos\left(\Omega t\right) & 0\\ 0 & 0 & 1 \end{array}\right]$.

From Equations (1) and (2), the range and range-rate (meter-meter/s) sequence can be obtained. To obtain the 2D coordinates in the range and cross-range domain (meter-meter):

(3) $$u\left(t\right)=r_{s}\left(t\right)=\left[\begin{array}{ccc} \cos\theta \cos\left(\Omega t-\varphi \right) & -\cos\theta \sin\left(\Omega t-\varphi \right) & \sin\theta \end{array}\right]{\vec{P}}_{s}$$

(4) $$v\left(t\right)=\frac{{\dot{r}}_{s}\left(t\right)}{\Omega \cos\theta }=\left[\begin{array}{ccc} -\sin\left(\Omega t-\varphi \right) & -\cos\left(\Omega t-\varphi \right) & 0 \end{array}\right]{\vec{P}}_{s}$$

One can note that Equations (3) and (4) are the projection equations in range and cross-range dimensions, respectively. For the convenience in the following discussion, the corresponding range projection vector and cross-range projection vector are defined as follows:

(5) $$\left\{ \begin{array}{l} {\vec{i}}_{t}=\left[\begin{array}{ccc} \cos\theta \cos\left(\Omega t-\varphi \right) & -\cos\theta \sin\left(\Omega t-\varphi \right) & \sin\theta \end{array}\right]\\ {\vec{j}}_{t}=\left[\begin{array}{ccc} -\sin\left(\Omega t-\varphi \right) & -\cos\left(\Omega t-\varphi \right) & 0 \end{array}\right] \end{array}\right.$$

Furthermore, note that the two vectors meet the following conditions:

(6) $$\{\begin{array}{c} \left\|{\vec{i}}_{t}\right\|=1\\ \left\|{\vec{j}}_{t}\right\|=1\\ {\vec{i}}_{t}⊥{\vec{j}}_{t} \end{array}$$

According to Equations (3)–(5), the projection can be written in matrix form as follows:

(7) W=MP

where the motion matrix $M={\left[{\vec{i}}_{1},\ldots ,{\vec{i}}_{M};{\vec{j}}_{1}\ldots {\vec{j}}_{m}\right]}^{T}$, shape matrix $P=\left[{\vec{P}}_{1},\ldots ,{\vec{P}}_{S}\right]$, and the projection matrix or the measurement matrix is:

(8) $$W=\left[\begin{array}{cccc} u_{11} & u_{12} & \cdots & u_{1S}\\ \vdots & \vdots & \ddots & \vdots \\ u_{M1} & u_{M2} & \cdots & u_{MS}\\ v_{11} & v_{12} & \cdots & v_{1S}\\ \vdots & \vdots & \ddots & \vdots \\ v_{M1} & v_{M2} & \cdots & v_{MS} \end{array}\right]$$

According to Equation (6), Equation (7) is an orthographic projection and the measurement matrix is the product of the motion matrix $M\in {\mathbb{R}}^{2M\times 3}$ and the shape matrix $P\in {\mathbb{R}}^{3\times S}$. Here, this paper focuses on recovering the space spinning target shape matrix ***I*** from the measurement matrix ***W*** via the factorization method [18]. To begin the 3D imaging procedure, the first step is to extract the scattering centers range and range-rate data set, which will be discussed in the next section. 

## 3. Feature Extraction

In this section, the feature extraction process will be discussed. Suppose the radar transmits wideband linear frequency modulation (LFM) signal and the echo signal can be written as:

(9) $$E\left(t_{m},f\right)=\sum_{s=1}^{S}A_{s}rect\left(\frac{f}{B}\right)rect\left(\frac{t_{m}}{T_{a}}\right)\exp\left[-j\frac{4\pi }{c}\left(f_{c}+f\right)R_{s}\left(t_{m}\right)\right]$$

where $R_{s}\left(t_{m}\right)=R_{0}\left(t_{m}\right)+r_{s}\left(t_{m}\right),\;R_{0}\left(t_{m}\right)$ is the radar-target range, $rect\left(u\right)=\{\begin{array}{cc} 1 & \left|u\right|\leq \frac{1}{2}\\ 0 & \left|u\right|>0 \end{array},\;\;B=\gamma \tilde{t}$ is the signal bandwidth, *γ* is the frequency modulation rate, $\tilde{t}$ is the fast time, *f_c_* is the carrier frequency, *T_a_* is the observation time window, *t_m_* is the slow time, *c* is the light speed and *A_s_* is the well-known reflectivity coefficient. Generally, the scattering coefficient is a complex function of frequency and radar look-angle. However, considering the narrow-angle measurement here, assume the backscattered intensity of each scatterer does not vary with the frequency and the view angle.

For the targets in high speed motion, the variation of radar-target range $R_{0}\left(t_{m}\right)$ in the fast time, together with the residual video phase (RVP) terms, results in distorted and widened high resolution range profiles (HRRPs). Therefore, coherent pre-processing is carried out to eliminate their effects. After the coherent pre-processing method [25] is applied to the ISAR raw data:

(10) $$E_{1}\left(t_{m},f\right)=\sum_{k=1}^{S}A_{s}rect\left(\frac{f}{B}\right)rect\left(\frac{t_{m}}{T_{a}}\right)\exp\left[-j\frac{4\pi }{c}\left(f_{c}+f\right)r_{s}\left(t_{m}\right)\right]$$

The instantaneous range induced by spinning motion could then be achieved using FT on Equation (10). However, to realize the enhanced resolution, the 2D spectral estimation algorithm presented in [22] is used to extract the range and the range-rate data. Therefore, Equation (10) can be rewritten in discrete form as follows:

(11) $$E_{1}\left(m,n\right)=\sum_{s=1}^{S}A_{s}\exp\left(-j4\pi \left(f_{0}+n\Delta f\right)\left(r_{s}+{\dot{r}}_{s}m\Delta t\right)/c\right)$$

where Δ*t* is the pulse repetition time, *r_s_* and ${\dot{r}}_{s}$ are the range and range rate of the scattering center *P_s_* at time zero, respectively. *n =* 0,…, *N* − 1, *N* is the sample number of the LFM pulse in the fast-time domain, Δ*f* = *B/N*, *f*_0_ = *f_c_*− *B*/2, *m* = 0,…, *M_hit_*− 1, *M_hit_* is the total number of coherent process hits. One can notice that the cross product $j4\pi mn\Delta f\Delta t{\dot{r}}_{s}$/*c* of the exponent in Equation (11) keeps it from conforming to the state space signal structure $\sum_{s}A_{s}^{\prime }\exp(jm\alpha_{s}+jn\beta_{s})$. To remove the cross product $j4\pi mn\Delta f\Delta t{\dot{r}}_{s}/c$, a new time-sampling interval Δ*t*’ satisfying (*f*_0_ + *n*Δ*f*)Δ*t* = *f*_0_Δ*t*’ is chosen, then the data matrix can be expressed as:

(12) $$E_{2}\left(m,n\right)=\sum_{s}A_{s}^{\prime }\exp\left(jm\alpha_{s}\right)\exp\left(jn\beta_{s}\right)$$

where $\alpha_{s}=4\pi f_{0}{\dot{r}}_{s}\Delta t/c,\;\beta_{s}=4\pi r_{s}\Delta f$/c, $A_{s}^{\prime }=A_{s}\exp\left(j4\pi f_{0}r_{s}/c\right)$. Now Δ*t* is the original time sampling interval and the ranges of both *m* and *n* over the rows and columns of ***E***_2_ are centered on zero. After transforming the measured data array ***E***_2_ in a form of the output of two coupled eigenvalue problems, the estimation result ${\hat{\alpha }}_{s}$ and ${\hat{\beta }}_{s}$ of the parameters can be straightforwardly determined. The amplitude estimation can be evaluated by a “least squares” procedure applied to (10), the ${\hat{\alpha }}_{s}$ and ${\hat{\beta }}_{s}$ now being known quantities. Then, the pairs provide a direct extraction of the range rate and the range:

(13) $${\hat{\dot{r}}}_{s}=\frac{c}{4\pi f_{0}\Delta t}{\hat{\alpha }}_{s}$$

(14) $${\hat{r}}_{s}=\frac{c}{4\pi \Delta f}{\hat{\beta }}_{s}$$

## 4. Scattering Center Association

After the acquisition of the ${\hat{r}}_{s}-{\hat{\dot{r}}}_{s}$ sequence from Equations (13) and (14), it is imperative that the sequence of ${\hat{r}}_{s}$ and ${\hat{\dot{r}}}_{s}$ estimates are associated with the same physical scattering centres. Note that the measurement matrix is the input of the factorization method. Therefore, a good scattering centres association is very important for the whole procedure, especially in low signal-to-noise ratio (SNR) environments. In [27], the scattering centres association is realised by exploiting minimum Euclidean distance criterion based on the Kalman filter (KF) [23] together with motion features. For the results presented here, the scattering centres association is realised based on the Kalman filter (KF) [23] and together with the scattering centres’ amplitude and motion features.

For a discrete linear Gaussian kinematic and measurement system, the KF provides a closed- form solution to estimate the measurement system state vector and covariance from the measurement histories. Without the loss of generality, the measurement models at time *t_m_* can be expressed as:

(15) $$x\left(m+1|m\right)=F_{m}x\left(m\right)+n_{x_{m}}$$

(16) $$z\left(m+1|m\right)=H_{m}x\left(m\right)+n_{z_{m}}$$

where ***x***(*m*) is the state vector, ***F****_m_* is the transition matrix of the system, ***z***(*m*) is the measurement vector, ***H****_m_* is the measurement matrix, $n_{x_{m}}$ and $n_{z_{m}}$ are the process noise and measurement noise, respectively. The index *m* means the index of the time sequence, so the estimated state vector can be given as:

(17) $$\hat{x}\left(m\right)=F_{m}\hat{x}\left(m-1\right)+G_{m}\left(z\left(m\right)-H_{m}F_{m}\hat{x}\left(m-1\right)\right)$$

where ***G**_m_* is the Kalman gain.

Here, the KF is used to estimate the scattering centres’ motion state vector and covariance from measurement histories. In the absence of noise:

(18) $$\hat{x}\left(m+1|m\right)=F_{m}\hat{x}\left(m\right)$$

From Equations (17) and (18), the prediction can be written as:

(19) $$\hat{x}\left(m+1|m\right)=F_{m}\hat{x}\left(m|m-1\right)+F_{m}G_{m}\left(z\left(m\right)-H_{m}F_{m}\hat{x}\left(m-1\right)\right)$$

where:

(20) $$F_{m}G_{m}=F_{m}\Psi_{m/m-1}H_{m}^{T}{\left[F_{m}\Psi_{m/m-1}H_{m}^{T}+\Gamma_{m}\right]}^{-1}$$

and the estimation error covariance matrix is:

(21) $$\Psi_{m/m-1}=F_{m}\Psi_{m-1}F_{m}^{T}+\Pi_{m-1}$$

where ***F*** and ***Π*** are the covariance matrixes of the measurement noise and the process noise, respectively. 

Considering the application here, it’s sufficient to describe the scattering center motion feature by the state vector which consists of the scattering centers range, range-rate and acceleration, i.e.,

(22) $$x\left(m\right)={\left[\begin{array}{ccc} r & \dot{r} & \ddot{r} \end{array}\right]}^{T}$$

We assume that the transition matrix is time-invariant, i.e., $F_{m}=F=\left[\begin{array}{ccc} 1 & T & 0.5T^{2}\\ 0 & 1 & T\\ 0 & 0 & 1 \end{array}\right],$ ***H****_m_* = ***H*** = [1 1 0], $n_{z_{x}}\sim N\left(0,\Gamma \right),\;\Gamma =\sigma_{z}^{2}I_{3},$ ***I***_3_ is the identity matrix. $n_{x_{m}}\sim$ ***N***(0,***Π***) with $\Pi =\left[\begin{array}{ccc} T^{4}/4 & T^{3}/2 & 0.5T^{2}\\ T^{3}/2 & T^{2} & T\\ T^{2}/2 & T & 1 \end{array}\right]\sigma_{x}^{2}$, where *T* is the pulse repetition time, $\sigma_{x}^{2}$ and $\sigma_{z}^{2}$ are the variance of the Gaussian noise.

Now, the approach for scattering centers trajectory tracking is given in detail. Let the number of the slow time samples be *M_a_*, so the range index *m* ∈ [1, *M_a_*]:

Step 1: Initialization. Let m = 1.
Step 2: For the *m*-th range and range-rate, we have the measurements $\left\{A_{\iota ,m},r_{\iota ,m},{\dot{r}}_{\iota ,m}\right\}$, where ι is the scattering center track index, $A_{\iota ,m}$ is the amplitude estimate. Define the initial measurements as $\left\{A_{\iota ,0},r_{\iota ,0},{\dot{r}}_{\iota ,0}\right\}$.

We assume that the transition matrix is time-invariant, i.e.: $F_{m}=F=\left[\begin{array}{ccc} 1 & T & 0.5T^{2}\\ 0 & 1 & T\\ 0 & 0 & 1 \end{array}\right],$ ***H****_m_* = ***H*** = [1 1 0], $n_{z_{x}}\sim N\left(0,\Gamma \right),\;\Gamma =\sigma_{z}^{2}I_{3},$ ***I***_3_ is the identity matrix. $n_{x_{m}}$ ~ ***N***(0,***Π***) with $\Pi =\left[\begin{array}{ccc} T^{4}/4 & T^{3}/2 & 0.5T^{2}\\ T^{3}/2 & T^{2} & T\\ T^{2}/2 & T & 1 \end{array}\right]\sigma_{x}^{2}$, where *T* is the pulse repetition time, $\sigma_{x}^{2}$ and $\sigma_{z}^{2}$ are the variance of the Gaussian noise.

Now, the approach for scattering centers trajectory tracking is given in detail. Let the number of the slow time samples be *M_a_*, so the range index *m* ∈ [1, *M_a_*]:

Step 1: Initialization*.* Let *m* = 1.
Step 2: For the m-th range and range-rate, we have the measurements $\left\{A_{i,m},r_{i,m},{\dot{r}}_{i,m}\right\}$, where *i* is the scattering center track index, *A_i,m_* is the amplitude estimate. We define the initial measurements as $\left\{A_{i,0},r_{i,0},{\dot{r}}_{i,0}\right\}$.
Step 3: Let *m* = *m* + 1. For the *i*-th scattering center track, search for the candidate scattering centers within the search window centered at *r_i,m__−_*_1_, then record the candidate set $\left\{A_{i,m}^{\prime },r_{i,m}^{i},{\dot{r}}_{i,m}^{\prime }\right\}$. Here, for a small observation interval, we assume the amplitude difference between the adjacent observation times is very small, so the optimal candidate for the *i*-th track can be determined by:
  (23) $$\left\{A_{\iota ,m},r_{\iota ,m},{\dot{r}}_{\iota ,m}\right\}=\arg\underset{\left({\hat{A}}_{\iota ,m}^{\prime },{\hat{r}}_{\iota ,m}^{\prime },{\hat{\dot{r}}}_{\iota ,m}^{\prime }\right)}{\min}{\left\|A_{\iota ,m}^{\prime }-A_{\iota ,m-1}\right\|}_{2}$$
  The other tracks are similarly associated with the scattering centers according to Equation (23). The acceleration of the scattering centers can be calculated as follows:
  (24) $${\ddot{r}}_{\iota ,0}=\frac{1}{T}\left({\dot{r}}_{\iota ,m}-{\dot{r}}_{\iota ,m-1}\right)$$
  Now, the initial state vector can be denoted as $x\left(0\right)={\left[r_{i,0}\;{\dot{r}}_{i,0}\;{\ddot{r}}_{i,0}\right]}^{T}$. Then, the next state $\left\{{\hat{x}}_{i,m+1/m}\right\}$ can be obtained according to Equations (19) to (21).
Step 4: Move to the next time index and let m=m+1. According to the minimum Euclidean distance criterion [23], the optimal candidate of the ιth scattering center track for the *m*-th range can be obtained by:
  (25) $$\left\{A_{\iota ,m},r_{\iota ,m},{\dot{r}}_{\iota ,m}\right\}=\arg\underset{\left(A_{\iota ,m}^{\prime },r_{\iota ,m}^{\prime },{\dot{r}}_{\iota ,m}^{\prime }\right)}{\min}{\left\|\xi_{\iota ,m}^{\prime }-\xi_{\iota ,m/m-1}\right\|}_{2}$$
  where $\left\{\xi_{i,m}^{\prime }\right\}$ is the feature set of the candidate scattering centers within the gating area and $\xi_{i,m/m-1}$ are the features of the predictions from the previous observables. $\xi_{\iota ,m}^{\prime }$ can be expressed as:
  (26) $$\xi_{\iota ,m}^{\prime }=\left[\mu_{1}A_{\iota ,m}^{\prime },\mu_{2}r_{\iota ,m}^{\prime },\mu_{3}{\dot{r}}_{\iota ,m}^{\prime }\right]$$
  where $\mu_{1}$, $\mu_{2}$ and $\mu_{3}$ are weight factors and $\mu_{1}+\mu_{2}+\mu_{3}=1$. According to Equations (23) and (26), Equation (25) can be rewritten as:
  (27) $$\left\{A_{\iota ,m},r_{\iota ,m},{\dot{r}}_{\iota ,m}\right\}=\arg\underset{\left(A_{\iota ,m}^{\prime },r_{\iota ,m}^{\prime },{\dot{r}}_{\iota ,m}^{\prime }\right)}{\min}\sqrt{{\left(\mu_{1}\frac{A_{\iota ,m}^{\prime }-A_{\iota ,m-1}}{A_{\iota ,m-1}}\right)}^{2}+{\left(\mu_{2}\frac{r_{\iota ,m}^{\prime }-{\hat{x}}_{\iota ,m/m-1}\left(1\right)}{{\hat{x}}_{\iota ,m/m-1}\left(1\right)}\right)}^{2}+{\left(\mu_{3}\frac{{\dot{r}}_{\iota ,m}^{\prime }-{\hat{x}}_{\iota ,m/m-1}\left(2\right)}{{\hat{x}}_{\iota ,m/m-1}\left(2\right)}\right)}^{2}}$$
  Finally, by using Equation (30), the scattering centres association is accomplished.
Step 5: Repeat Step 4 to finish the whole measurements association.

It should be noted that the difference among scattering centres near the intersection points is so small that incorrect associations may appear. In this case, the multistep prediction in Steps 3 and 4 can be performed to increase the separability. Within the gating area, the combinations of features for all the candidate scattering centres are used to calculate the Euclidean distance. For avoiding the jump at the intersection points, the predicted amplitude will be replaced by the average amplitude of the existing associated tracks. And then, the minimum one in Equation (25) is the optimal association. Here, the three-step prediction is taken as an example. Suppose the feature set of all the candidates is $\left\{\xi_{i,\left[k,k+2\right]}^{\prime }\right\}$, then Equation (27) can be rewritten as:

(28) $$\left\{A_{\iota ,k},r_{\iota ,k},{\dot{r}}_{\iota ,k}\right\}=\arg\underset{\left(A_{\iota ,k}^{\prime },r_{\iota ,k}^{\prime },{\dot{r}}_{\iota ,k}^{\prime }\right)}{\min}{\left\|\xi_{\iota ,\left[k,k+2\right]}^{\prime }-\xi_{\iota ,\left[k,k+2\right]}\right\|}_{2}$$

where:

(29) $$\xi_{\iota ,\left[k,k+2\right]}=\left[\mu_{1}{\bar{A}}_{\iota ,k-1},\mu_{2}\left[{\hat{x}}_{\iota ,k/k-1}\left(1\right),{\hat{x}}_{\iota ,k+1/k}\left(1\right),{\hat{x}}_{\iota ,k+2/k+1}\left(1\right)\right],\mu_{3}\left[{\hat{x}}_{\iota ,k/k-1}\left(2\right),{\hat{x}}_{\iota ,k+1/k}\left(2\right),{\hat{x}}_{\iota ,k+2/k+1}\left(2\right)\right]\right]$$

where ${\bar{A}}_{i,k-1}$ is the average amplitude of the existing associated i-th track. ${\hat{x}}_{i,\varsigma +1/\varsigma }\;\left(\varsigma =k-1,k,k+1\right)$ are the predictions. 

## 5. Factorization-Based 3D Imaging and Scaling

**Figure 2:**
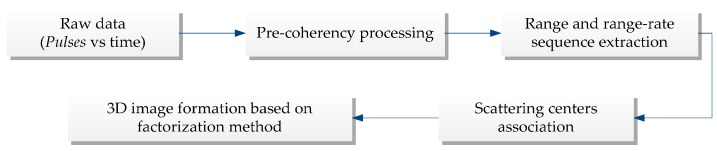
3D space spinning target imaging methodology.

Based on the above development, the general flow chart for processing a sequence of pulse echoes to form a 3D image is depicted in Figure 2. In this section, the 3D imaging procedure of the proposed method will be discussed in detail.

### 5.1. 3D Imaging Based on Factorization Method

Because the spinning angular velocity is unknown, according to Equation (4), an initial coarse angular velocity Ω_0_ is chosen to scale the cross-range to obtain the measurement matrix. The rank of the measurement matrix ***W*** is highly rank-deficient [19]. In a real scenario, the rank of ***W*** is not exactly three, but approximately three. Therefore, orthogonal matrices through SVD decomposition can be achieved as:

(30) $$W=\left[\begin{array}{cc} {\left(U_{1}\right)}_{2M\times 3} & {\left(U_{2}\right)}_{2M\times \left(S-3\right)} \end{array}\right]\left[\begin{array}{cc} {\left(\Sigma_{1}\right)}_{3\times 3} & 0\\ 0 & 0 \end{array}\right]\left[\begin{array}{c} {\left(V_{1}\right)}_{3\times S}\\ {\left(V_{2}\right)}_{\left(S-3\right)\times S} \end{array}\right]$$

So we can factorize ***W*** into:

(31) $$W=\hat{M}\hat{P}$$

where $\hat{M}=U_{1}{\left(\Sigma_{1}\right)}^{1/2}$ and $\hat{P}={\left(\Sigma_{1}\right)}^{1/2}V_{1}$.

Since the decomposition is not unique, the following step is to find an invertible matrix $\Delta \in {\mathbb{R}}^{3\times 3}$ and then the true solutions $\tilde{M}$ and $\tilde{P}$ can be obtained as follows:

(32) $$\tilde{M}=\hat{M}\Delta$$

(33) $$\tilde{P}=\Delta^{-1}\hat{P}$$

According to the corresponding constraints in Equation (6), we obtain the system of 3M overdetermined equations, such that:

(34) $$\begin{array}{c} {\hat{i}}_{m}^{T}L{\hat{i}}_{m}=1\\ {\hat{j}}_{m}^{T}L{\hat{j}}_{m}=1\\ {\hat{i}}_{m}^{T}L{\hat{j}}_{m}=0 \end{array}$$

where ${\hat{i}}_{m}$ and ${\hat{j}}_{m}$ are the mth row and the (*M* + *m*)-throw of $\hat{M}$, respectively. *L* = *ΔΔ^T^* is the symmetric matrix:

(35) $$L=\left[\begin{array}{ccc} l_{1} & l_{2} & l_{3}\\ l_{2} & l_{4} & l_{5}\\ l_{3} & l_{5} & l_{6} \end{array}\right]$$

Equation (34) can be rewritten as:

(36) Θl=χ

where $\Theta \in {\mathbb{R}}^{3M\times 6}$, $l\in {\mathbb{R}}^{6\times 1}$, and $\chi \in {\mathbb{R}}^{3M\times 1}$ are defined by:

(37) $$\Theta =\left[\begin{array}{c} g^{T}\left(i_{1},i_{1}\right)\\ \vdots \\ g^{T}\left(i_{M},i_{M}\right)\\ g^{T}\left(j_{1},j_{1}\right)\\ \vdots \\ g^{T}\left(j_{M},j_{M}\right)\\ g^{T}\left(i_{1},j_{1}\right)\\ \vdots \\ g^{T}\left(i_{M},j_{M}\right) \end{array}\right],l=\left[\begin{array}{c} l_{1}\\ \vdots \\ l_{6} \end{array}\right],\chi =\left[\begin{array}{l} \begin{array}{c} 1\\ \vdots \\ \vdots \\ \vdots \\ \vdots \\ 1 \end{array}\}2M\\ \begin{array}{c} 0\\ \vdots \\ 0 \end{array}\}M \end{array}\right]$$

And for two arbitrary vectors ***a*** = [*a*_1_ *a*_2_ *a*_3_] and ***b*** = [*b*_1_ *b*_2_ *b*_3_], we define:

(38) $$g^{T}\left(a,b\right)=\left[a_{1}b_{1},a_{1}b_{2}+a_{2}b_{1},a_{1}b_{3}+a_{3}b_{1},a_{2}b_{2},a_{2}b_{3}+a_{3}b_{2},a_{3}b_{3}\right]$$

So $\hat{l}$ can be solved by the pseudo-inverse method, that is:

(39) $$\hat{l}={\left(\Theta^{T}\Theta \right)}^{-1}\Theta^{T}\chi$$

Then, the symmetric matrix ***L*** can be constructed using $\hat{l}$. Applying eigenvalue decomposition to ***L***:

(40) $$L=B\Lambda B^{T}$$

where ***B*** and ***Λ*** are the eigenvector matrix and the diagonal eigenvalue matrix, respectively. Then, the solutions for the invertible matrix can be obtained as:

(41) $$\Delta =B\Lambda^{1/2}$$

Finally, $\tilde{M}$ and $\tilde{P}$ can be obtained by substituting Equation (41) into Equations (32) and (33), respectively. 

### 5.2. 3D Image Scaling

Although the 3D image of the target can be obtained according to Equation (33), the image is modified by a scaling factor. This is because the cross-range is scaled by the coarse spinning angular velocity $\Omega_{0}$ and the scale ambiguities in cross-range will result in ambiguities in the spatial axes of the reconstructed target geometry. In this section, the image scaling process is introduced to remove these ambiguities.

**Figure 3:**
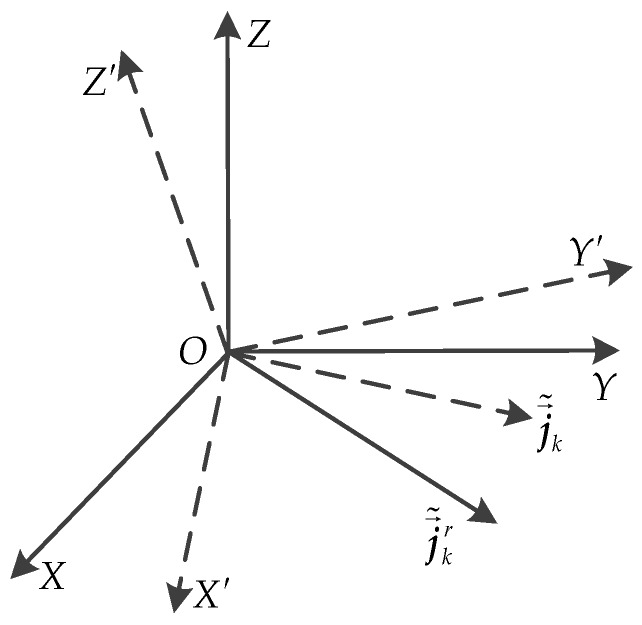
The new coordinate system *O-X’Y’Z’* after 3D rotation.

Comparing to the real 3D geometry and projection matrix, it is worth noting that the estimated result $\tilde{M}$ and $\tilde{P}$ obtained via Equations (32) and (33) are the rotated projection matrix and 3D geometry matrix, respectively. Because the real 3D geometry and motion matrix is expressed in *O-XYZ*, suppose the rotated coordinate system is *O-X’Y’Z’*, as shown in Figure 3.

The real 3D geometry estimate ${\tilde{P}}_{r}$ and motion matrix estimate ${\tilde{M}}_{r}$ can be obtained via the rotation operation, i.e.,

(42) $${\tilde{M}}_{r}=\tilde{M}Q^{T}$$

(43) $${\tilde{P}}_{r}=Q\tilde{P}$$

where ***Q*** is the matrix with ***Q***^T^***Q*** = ***QQ***^T^ = ***I***_3__×__3_, ${\tilde{M}}_{r}={\left[{\tilde{\vec{i}}}_{1}^{r},\ldots ,{\tilde{\vec{i}}}_{M}^{r};{\tilde{\vec{j}}}_{1}^{r},\ldots ,{\tilde{\vec{j}}}_{M}^{r}\right]}^{T}$, ${\tilde{P}}_{r}=\left[{\tilde{\vec{P}}}_{1}^{r},\ldots ,{\tilde{\vec{P}}}_{s}^{r}\right]$. According to [24], the matrix ***Q*** can be represented by three continuous rotations around the three coordinate axes. We define the rotational angles around the three axes as *φ_x_*, *φ_y_* and *φ_z_*, respectively, that is:

(44) $$Q=R_{z}R_{y}R_{x}$$

where:

$$R_{z}=\left[\begin{array}{ccc} \cos\psi_{z} & \sin\psi_{z} & 0\\ -\sin\psi_{z} & \cos\psi_{z} & 0\\ 0 & 0 & 1 \end{array}\right],\;R_{y}=\left[\begin{array}{ccc} \cos\psi_{y} & 0 & -\sin\psi_{y}\\ 0 & 1 & 0\\ \sin\psi_{y} & 0 & \cos\psi_{y} \end{array}\right],\;R_{x}=\left[\begin{array}{ccc} 1 & 0 & 0\\ 0 & \cos\psi_{x} & \sin\psi_{x}\\ 0 & -\sin\psi_{x} & \cos\psi_{x} \end{array}\right]$$

According to Equation (6), ${\tilde{\vec{j}}}_{m}$ is perpendicular to the $OZ^{\prime }$ axis, as shown in Figure 3. After the rotation, ${\tilde{\vec{j}}}_{m}^{r}$ should be still perpendicular to the *OZ* axis, i.e., ${\tilde{\vec{j}}}_{m}^{r}\left(3\right)=0$, so ***Q*** can be estimated via solving the optimization problem below:

(45) $$\left(\psi_{x},\psi_{y},\psi_{z}\right)=\arg\min\left\{{\left[{\left(j^{r}\right)}_{col,3}\right]}^{T}{\left(j^{r}\right)}_{col,3}\right\}$$

where $j^{r}=\left[{\tilde{\vec{j}}}_{1}^{r},\ldots ,{\tilde{\vec{j}}}_{M}^{r}\right]$, and ${\left(j^{r}\right)}_{col,3}$ represents the third column of *j^r^*.

The optimization problem in Equation (45) is nonlinear and can be solved by exhaustive search. After ***Q*** is formed, ${\tilde{M}}_{r}$ and ${\tilde{P}}_{r}$ can be obtained via Equations (42) and (43). A new vector can be formulated as follows:

(46) $$\begin{array}{ll} h\left(m\right) & ={{\tilde{\vec{j}}}_{m}}^{r}\left(2\right)+j\cdot {{\tilde{\vec{j}}}_{m}}^{r}\left(1\right)\\ & =\exp\left(j\left(\Omega t_{m}+\varphi_{c}\right)\right) \end{array}$$

where *φ_c_* is the constant angle, which is an unimportant constant factor. The phase of Equation (46) can be written as:

(47) $$∠h\left(m\right)=m\Omega T+\varphi_{c}$$

Due to the phase *φ_c_* or the fast spinning angular velocity, during the observation time, phase ambiguity may occur. The ambiguity can be removed by judging the continuity of $∠h\left(m\right)=m\Omega T+\phi_{c}$. Then by extracting the polynomial coefficients $\hat{\eta }$ of the phase, the estimate of the angular velocity can be obtained:

(48) $$\hat{\Omega }=\frac{\left|\hat{\eta }\right|}{T}$$

Based on the estimated cross-range resolution, scaling of the cross-range coordinates in ***W*** is achieved. After that, the factorization method is performed to ***W*** again. The scaling and factorization steps will be carried out iteratively until the value of Equation (45) is smaller than a threshold $\varepsilon_{0}$. The steps of the proposed method are described in Algorithm 1.

**Algorithm 1:** Processing steps of the proposed method**Input**: Raw data**Pre-processing**: -Apply the pre-coherency processing [25] to the data -Extract the range and range-rate using the spectral estimate method [22] -Correlate the range and range-rate sequenceusing method developed in Section 4**Initialization**: initialize *k* = 0, and choose an initial coarse angluar velocity ${\hat{\Omega }}_{0}$ and the error threshold $\varepsilon_{0}$. Perform scaling to the sequential range-rate to form the projection matrix ***W***_0_ according to Equations (3), (4) and (8)**Main iteration**: increase *k* by 1 and perform the following procedure:Step 1: Submit ${\hat{\Omega }}_{0}$ to Equation (4) to rescale the cross-range and obtain the scaled matrix ***W**_k_*Step 2: Calculate the motion matrix ${\hat{M}}_{k}$ and shape matrix ${\hat{P}}_{k}$ by substituting ***W****_k_* into Equations (32) and (33) Step 3: According to Equations (42)−(45) and the result in Step 2, calculate the rotational transform matrix ***Q****_k_*Step 4: Estimate the angular velocity ${\hat{\Omega }}_{k}$ by substituting ***Q****_k_* into Equations (46)−(48)Step 5: If $\left({\left[{\left(j^{r}\right)}_{col,3}\right]}^{T}{\left(j^{r}\right)}_{col,3}\right)<\varepsilon_{0}$, then ${\hat{\Omega }}_{k}={\hat{\Omega }}_{true}$, and stop the iteration. Otherwise, repeat the main iteration.**Output**: Estimate ${\hat{\Omega }}_{true}$. Consequently, the well-scaled shape matrix ${\hat{P}}_{r}$ can be generated using the optimal ${\hat{\Omega }}_{true}$.

## 6. Simulation Results

### 6.1. Simulation Results

**Figure 4:**
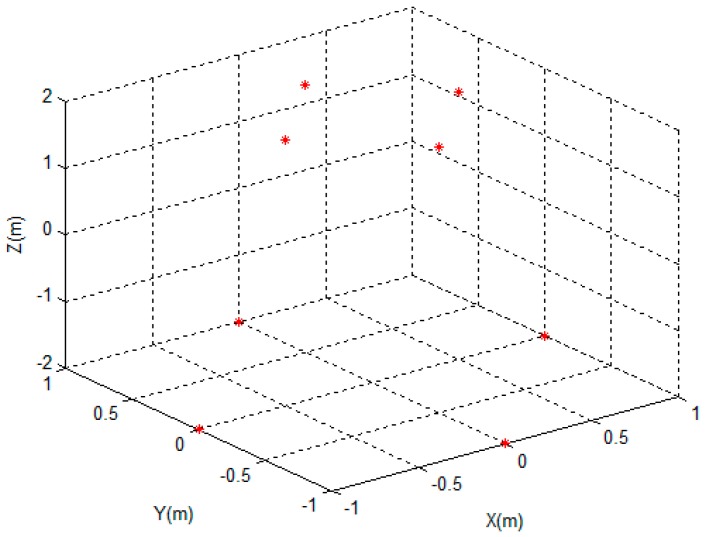
The distribution of the scattering centers.

In this section, the performance of the proposed method is verified using the point scattering centre model. As shown in Figure 4, we suppose the 3D target consists of eight scattering centres. The coordinates and the scattering coefficients are listed in Table 1. 

The radar signal centre frequency is 10 GHz and the bandwidth is 1 GHz, giving a range resolution of 0.15 m. The pulse repetition frequency is 100 Hz and the whole observation time is 1 s. Suppose the initial Euler angles are 30°, 20° and 50°, respectively. The elevation and azimuth angle of the RLOS are both 45°. The target spinning frequency is 2 Hz. The pulse time width is 0.3 ms and the dechirping signal sampling is rate 40 ZMHz. In this simulation, Gaussian noise is added to the simulated echo signal and the SNR is 10 dB.

**Figure 5:**
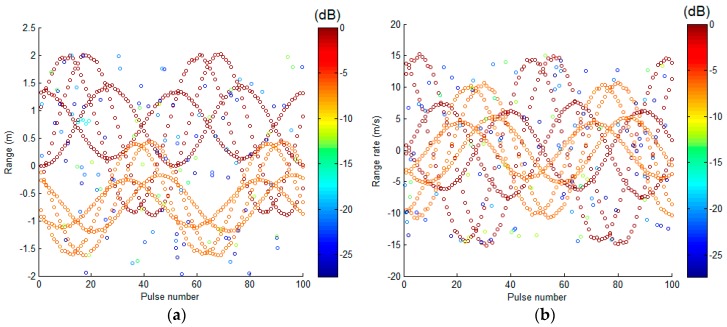
1D range and range rate estimates: (**a**) range estimate; (**b**) range rate estimate.

Before using the spectrum analysis method [18] to extract the range and range-rate data, the coherent pre-processing [25] is performed on the echoes in advance. As a result, Figure 5a,b illustrate the sequential range and range-rate estimates, respectively. The colors in Figure 5 depict the normalized scattering coefficient of the scattering centers. Note that the scattering centers are clearly resolved in range and range-rate. 

**Figure 6:**
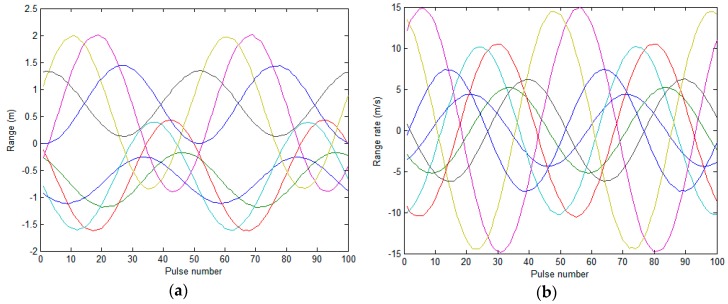
1D range and range rate data association result: (**a**) 1D range data association result; (**b**) range rate data association result.

To obtain the measurement matrix ***W*** in Equation (8), the scattering center range and range-rate data set needs to be associated. Figure 6 presents the association results of the range and range-rate sequence using the method developed in Section 4. Here, the weight factors *μ*_1_, *μ*_2_, *μ*_3_ are set as 0.4, 0.4 and 0.2, respectively. The correlated scattering centers correspond to the same color.

After finishing the extraction and tracking of the eight scattering centers, the initial coarse value of the angular velocity is set to 10 rad/s to form the projection matrix ***W***_0_ according to Equations (3), (4) and (8).

**Figure 7:**
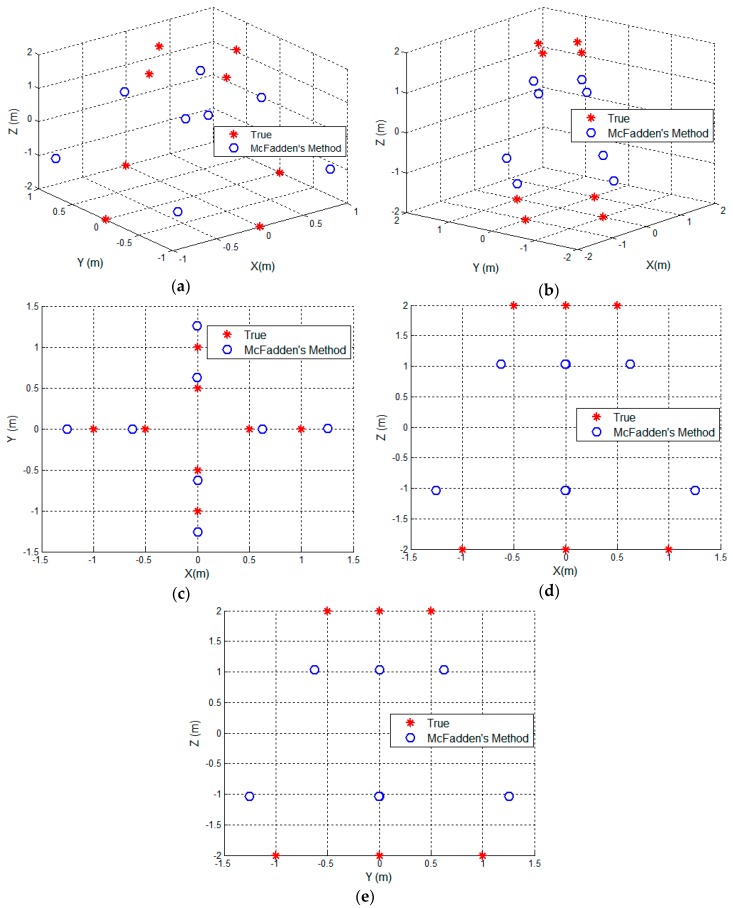
The reconstructed result using McFadden’s method [20]. (**a**) The distribution of the reconstructed 3D scattering centers before transformation; (**b**) The distribution of the 3D scattering centers after transformation; (**c**) the distribution on *XY* plane; (**d**) the distribution on *XZ* plane; (**e**) the distribution on *YZ* plane.

The reconstructed 3D geometry using the McFadden’s method [20] is presented in Figure 7. The blue circles represent the reconstructed scattering centers. From Figure 7a, one can find there are transformation and scale ambiguities between the reconstructed target and the true one. This is because the cross-range in the projection matrix is not accurate. By using McFadden’s method [20], the ambiguity of the cross-range will result in the scale ambiguities of the reconstructed scattering centers position. 

After the transformation, Figure 7b–e shows the distribution of the reconstructed scattering centers in 3D space, *XY* plane, *XZ* plane and *YZ* plane, respectively. From Figure 7b–e, one can find the apparent scale ambiguities in the 3D dimensions. 

**Figure 8:**
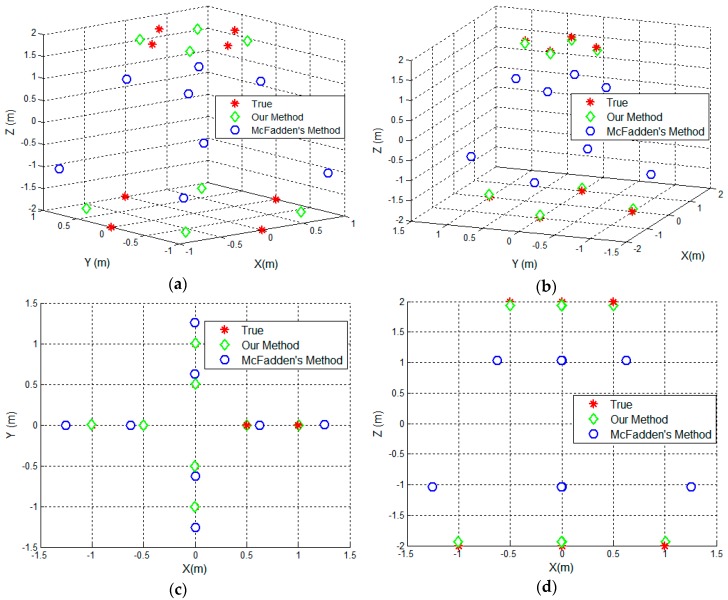

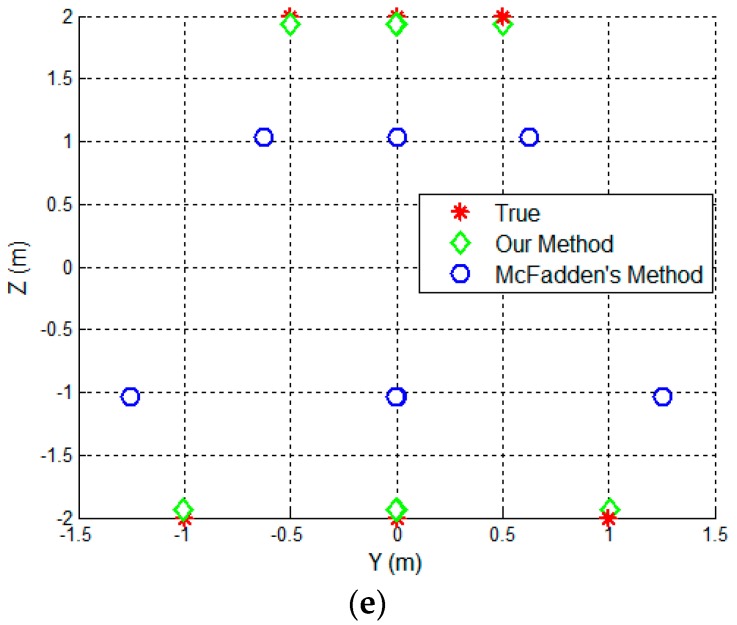
The reconstructed result using the proposed method. (**a**) The distribution of the reconstructed 3D scattering centers before transformation; (**b**) The distribution of the 3D scattering centers after transformation; (**c**) the distribution on *XY* plane; (**d**) the distribution on *XZ* plane; (**e**) The distribution on *YZ* plane.

To remove the ambiguities shown in the Figure 7, the proposed scaling algorithm is implemented. Figure 8a shows the final reconstructed results using the proposed scaling algorithm and McFadden’s method, respectively. Here, the error threshold $\varepsilon_{0}$ or the iteration procedure is set to 5e-2. After the transformation, the coordinates of the scattering centers are listed in Table 2. Figure 8b–e show the distribution of the reconstructed scattering centers in 3D space, *XY* plane, *XZ* plane and *YZ* plane. As shown in Figure 8b–e, the reconstructed target coincides well with the true one. Comparing to the reconstructed result via McFadden’s method, one can find the ambiguity has been removed by using the proposed scaling algorithm. The reconstruction result in Figure 8 proves the effectiveness of the proposed method.

### 6.2. Performance Analysis

To evaluate the performance of the proposed algorithm quantitatively, the root mean square error (RMSE) of the recovered 3D target geometry is calculated in the terms of Euler distance error:

(49) $$RMSE\left(P\right)=\sqrt{E\left[{\left(\hat{P}-P\right)}^{T}\left(\hat{P}-P\right)\right]}$$

The RMSE of the estimate of the spinning angular velocity is defined as:

(50) $$RMSE\left(\Omega \right)=\sqrt{E\left[{\left|\hat{\Omega }-\Omega \right|}^{2}\right]}$$

where *E*[*X*] denotes the average of the *X*.

#### 6.2.1. Effect of the SNR Level and Pulse Quantity

**Figure 9:**
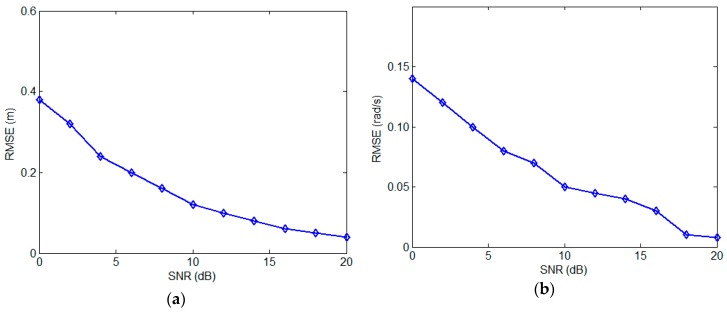
RMSE with respect to SNR. (**a**) 3D geometry; (**b**) spinning angular velocity.

We note that the proposed algorithm performance is mainly affected by two factors: the noise level and the quantity of the pulses. Therefore, the experiments are designed to analyze the two factors. The first experiment is to analyze the effects of different SNR level on the algorithm performance. In this experiment, the pulse number is fixed at 100 and the SNR level varies from 0 to 20 dB. For each SNR, the experiment is carried out with 500 Monte-Carlo simulations, and the two RMSE curves against SNR are presented in Figure 9. As shown in Figure 9, with the increasing of SNR, the RMSEs of the two parameters decrease and low SNR level has an obvious impact on the 3D reconstruction and angular velocity estimation performance.

**Figure 10:**
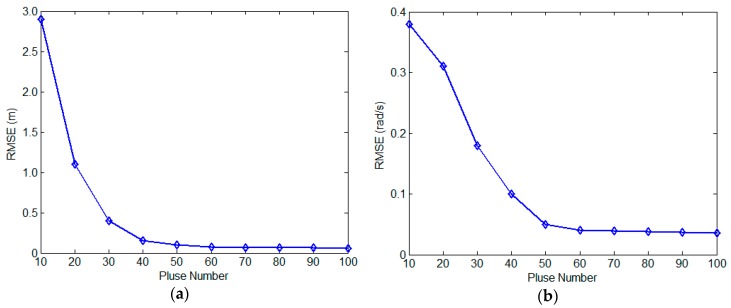
RMSE against pulse number. (**a**) 3D geometry; (**b**) spinning angular velocity.

In the second experiment, we test the algorithm performance by varying the quantity of the pulses. The SNR is set to 10 dB and the echo pulse number varies from 10 to 100 in steps of 10. The experiment is also carried out with 500 Monte-Carlo simulations for each pulse number, and the RMSE curves against pulse quantity is described in Figure 10. As shown in Figure 10, it can be found that when the pulse quantity is less than 30, both of the RMSEs of the two parameters decrease quickly with the increment of the pulse number. Therefore, more pulses will benefit the robustness of the proposed method. Meanwhile, when the pulse number is more than 50, there’s little difference in performance. This is because the target spinning angle is over 360° when the pulse number is over 50 and the information for reconstruction is abundant. From Figure 9 and Figure 10, we can draw the conclusion that the large number of utilized pulses and accurate extraction and tracking will bring good performance of the proposed method.

#### 6.2.2. Effect of Coarse Initial Angular Velocity

**Figure 11:**
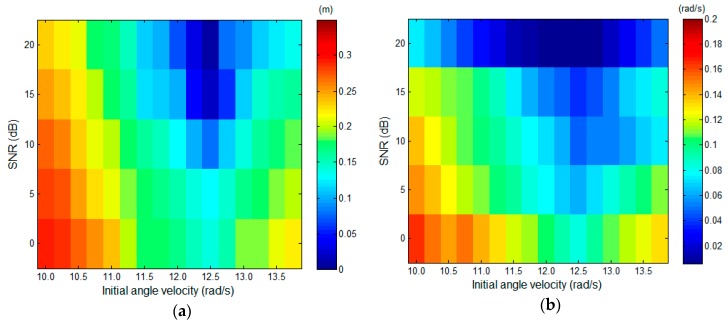
RMSEs of 3D reconstruction and angular velocity estimation. (**a**) RMSE of the 3D reconstruction against different initial angular velocity and SNR; (**b**) RMSE of the angular velocity estimate against different initial angular velocity and SNR.

To analyze the impact of the different initial angular velocity values on the 3D imaging and angular velocity estimation, here another experiment is designed. In this experiment, the coarse angular velocity used to start the reconstruction procedure varies from 10.0 rad/s to 13.5 rad/s. The number of the pulse used for imaging is 100. The SNR level varies from 0 to 20 dB with a step of 5 dB. For each SNR level and initial angular velocity, 500 Monte-Carlo simulations are carried out. Figure 11 presents the RMSEs calculation result of the 3D reconstruction and angular velocity estimate. The largest RMSEs of 3D imaging and angular velocity estimation are 0.34 m and 0.16 rad/s, respectively which are acceptable. From Figure 9a,b, one can find that RMSEs decrease with the initial coarse value getting closer to the true value. And The RMSEs of the two estimates are minimized when the initial angular velocity is equal to the true value. Therefore, the precise initial angular velocity will benefit the target 3D imaging.

## 7. Conclusions

In this paper, a 3D imaging and scaling algorithm for rapidly spinning target based on factorization method is proposed. Due to the lack of freedom, the recovered 3D imaging of the spinning target via traditional factorization method is a rotated and scaled version of the true one. The proposed method provides a new solution for 3D imaging of spinning targets and removes the scale ambiguities in the recovered 3D image. Simulation results show the effectiveness and robustness of the proposed algorithm. In the future, we will test the algorithm with real measured data.

## Figures and Tables

**Table 1:** Parameters of the scattering centers.

Scattering Centre Index	*X*	*Y*	*Z*	Scattering Coefficient
#1	0.5	0	2	1
#2	−0.5	0	2	1
#3	0	0.5	2	1
#4	0	−0.5	2	1
#5	1.0	0	−2	2
#6	−1.0	0	−2	2
#6	0	1.0	−2	2
#8	0	−1.0	−2	2

**Table 2:** Coordinates of reconstructed scattering centers.

Scattering Center Index	*X*	*Y*	*Z*
#1	0.5029	−0.0030	1.9316
#2	−0.5024	0.0016	1.9324
#3	0.0022	0.5023	1.9315
#4	0.0024	−0.5025	1.9317
#5	1.0048	0.0029	−1.9332
#6	−1.0048	0.0026	−1.9316
#6	0.0019	1.0052	−1.9306
#8	0.0025	−1.0043	−1.9323
